# TOP-Net Prediction Model Using Bidirectional Long Short-term Memory and Medical-Grade Wearable Multisensor System for Tachycardia Onset: Algorithm Development Study

**DOI:** 10.2196/18803

**Published:** 2021-04-15

**Authors:** Xiaoli Liu, Tongbo Liu, Zhengbo Zhang, Po-Chih Kuo, Haoran Xu, Zhicheng Yang, Ke Lan, Peiyao Li, Zhenchao Ouyang, Yeuk Lam Ng, Wei Yan, Deyu Li

**Affiliations:** 1 Key Laboratory for Biomechanics and Mechanobiology of Ministry of Education, Beijing Advanced Innovation Center for Biomedical Engineering, School of Biological Science and Medical Engineering Beihang University Beijing China; 2 Department of Computer Management and Application Chinese PLA General Hospital Beijing China; 3 Center for Artificial Intelligence in Medicine Chinese PLA General Hospital Beijing China; 4 Department of Biomedical Engineering Chinese PLA General Hospital Beijing China; 5 Laboratory for Computational Physiology Institute for Medical Engineering and Science Massachusetts Institute of Technology Cambridge, MA United States; 6 Medical School of Chinese PLA Beijing China; 7 US Research Lab PingAn Tech San Francisco, CA United States; 8 Beijing SensEcho Science & Technology Co., Ltd Beijing China; 9 Department of Computer Science and Technology Tsinghua University Beijing China; 10 Hangzhou Innovation Institute Beihang University Beijing China; 11 Faculty of Arts & Science University of Toronto Toronto, ON Canada; 12 Department of Hyperbaric Oxygen Chinese PLA General Hospital Beijing China

**Keywords:** tachycardia onset, early prediction, deep neural network, wearable monitoring system, electronic health record

## Abstract

**Background:**

Without timely diagnosis and treatment, tachycardia, also called tachyarrhythmia, can cause serious complications such as heart failure, cardiac arrest, and even death. The predictive performance of conventional clinical diagnostic procedures needs improvement in order to assist physicians in detecting risk early on.

**Objective:**

We aimed to develop a deep tachycardia onset prediction (TOP-Net) model based on deep learning (ie, bidirectional long short-term memory) for early tachycardia diagnosis with easily accessible data.

**Methods:**

TOP-Net leverages 2 easily accessible data sources: vital signs, including heart rate, respiratory rate, and blood oxygen saturation (SpO_2_) acquired continuously by wearable embedded systems, and electronic health records, containing age, gender, admission type, first care unit, and cardiovascular disease history. The model was trained with a large data set from an intensive care unit and then transferred to a real-world scenario in the general ward. In this study, 3 experiments incorporated merging patients’ personal information, temporal memory, and different feature combinations. Six metrics (area under the receiver operating characteristic curve [AUROC], sensitivity, specificity, accuracy, F1 score, and precision) were used to evaluate predictive performance.

**Results:**

TOP-Net outperformed the baseline models on the large critical care data set (AUROC 0.796, 95% CI 0.768-0.824; sensitivity 0.753, 95% CI 0.663-0.793; specificity 0.720, 95% CI 0.645-0.758; accuracy 0.721; F1 score 0.718; precision 0.686) when predicting tachycardia onset 6 hours in advance. When predicting tachycardia onset 2 hours in advance with data acquired from our hospital using the transferred TOP-Net, the 6 metrics were 0.965, 0.955, 0.881, 0.937, 0.793, and 0.680, respectively. The best performance was achieved using comprehensive vital signs (heart rate, respiratory rate, and SpO_2_) statistical information.

**Conclusions:**

TOP-Net is an early tachycardia prediction model that uses 8 types of data from wearable sensors and electronic health records. When validated in clinical scenarios, the model achieved a prediction performance that outperformed baseline models 0 to 6 hours before tachycardia onset in the intensive care unit and 2 hours before tachycardia onset in the general ward. Because of the model’s implementation and use of easily accessible data from wearable sensors, the model can assist physicians with early discovery of patients at risk in general wards and houses.

## Introduction

Tachycardia, a heart rhythm disorder, is defined as an adult resting heart rate that exceeds 100 bpm [1]. According to the mechanisms, causes, expressions and outcomes, tachycardia can be classified as sinus tachycardia, atrial fibrillation, atrial flutter, ventricular tachycardia, or ventricular fibrillation [2]. Spontaneous ventricular tachyarrhythmia is a major cause of sudden cardiac death; approximately 180,000 to 300,000 people suffer from this condition in the US yearly [3,4]. Atrial fibrillation is a risk factor for stroke, congestive heart failure, and premature death. Patients suffering from atrial fibrillation for the first time have a high rate of mortality [5,6]. In addition, tachycardia has been correlated to poor outcomes [7]. Conventional tachycardia detection depends on cardiologists or clinical experts reading electrocardiogram (ECG) signals. Due to limited numbers of measurements and the intermittent nature of the diseases, the symptoms of tachycardia might not be captured when ECGs are recorded in hospitals [8]. Therefore, continuous monitoring enables clinicians to early diagnose, predict the disease, and have enough time to prevent patients from deteriorating.

Recently, several hospitals have attempted to utilize wearable devices for continuous monitoring of vital signs such as heart rate, respiration rate, and oxygen saturation (SpO_2_) [9,10]. The adoption of wearable devices in hospitals facilitates the acquisition of patient status anywhere and anytime to reduce the workload of nurses. Compared with the use of single-threshold alarm monitoring devices and commonly used early warning scores defined by clinical experts [11], machine learning methods can automatically discover patterns and relationships within data without human instructions. Thus, machine learning has been proven as an effective clinical tool to identify abnormal events or provide early warning of diseases based on electronic health record, biomarker, gene expression, and imaging data [12-14]. Forkan et al [15] leveraged a hidden Markov model to predict 7 clinical onsets, including tachycardia onset, and further improved performance by using random forest algorithms to forecast events within 1 to 2 hours [16]. Lee et al [17] developed an artificial neural network to predict ventricular tachycardia within 1 hour. Szep et al [18] utilized an archetypal cardiac monitoring system with regression and boosting models to detect arrhythmia and predict the fatal arrhythmia several minutes before onset.

With nonlinear computation and flexible feature extraction, deep learning models show strong performances in representation learning and exploration of unknown information [19]. Researchers have recently used deep learning models for disease diagnosis and prediction based on physiological signals or electronic health records [20-22]. Since measuring and acquiring vital signs are easily measured and some open-source, labeled physiological signal (especially ECG signals) data sets are available [23,24], there exist many studies employing deep learning in cardiology [25]. Hannun et al [26] reported a convolutional neural network algorithm that detects heart arrhythmias using ECG signals acquired with a single-lead wearable sensor. Shashikumar et al [27] also presented a convolutional neural network model that detects and monitors atrial fibrillation. Teijeiro et al [28] introduced a long short-term memory (LSTM) network based on a set of features extracted from ECG records to classify normal sinus rhythm, atrial fibrillation, and anomalies. Gotlibovych et al [8] constructed a model combining a convolutional neural network and LSTM to achieve nearly real-time identification of atrial fibrillation. Cho et al [29] obtained a convolutional neural network model to predict atrial fibrillation within 4 to 6 minutes using ECG signals.

Cardiovascular diseases are complex and heterogeneous; multiple factors such as genetics, environment, age, and gender can affect the occurrence and severity of cardiovascular disease [30,31]. Age has been proven to be an independent risk factor, and being female is a greater risk factor for cardiovascular disease when elderly [31]. Few studies have attempted to develop a prediction tachycardia onset model that accounts for the patient’s personal information. Respiratory dysfunction and common lung diseases, such as asthma, chronic obstructive pulmonary disease, and lung fibrosis are significantly more likely to cause cardiovascular disease [32]. Abnormal respiratory rate and its relative changes are a critical indicator to predict cardiac arrest [33], and SpO_2_ has also been shown as a diagnostic marker of acute heart failure [34]. However, this useful information has not been used effectively, though it can be easily acquired with wearable sensors.

The aim of this study was to develop a bidirectional long short-term memory (BiLSTM) model—TOP-Net—that is applicable to both intensive care units and general wards [35], leverages easily accessible data, enables real-time evaluation and early prediction of tachycardia onset with a long forecast range, and is based on vital signs and electronic health record data with the following contributions: (1) combining electronic health record (sparse records) and biosensor data (high frequency records) to accomplish early prognosis and real-time prediction of tachycardia onset, and its performance of early prediction; (2) being the first to consider 2 other important vital signs and explore their different combinations being with deep learning models to predict tachycardia onset, which can improve the precision of early forecast; and (3) utilizing a large critical care data set and a model that is transferrable to real clinical scenarios wards where patients are monitored by medical-grade wearable embedded systems, for example, transferable between different countries (US to China), ethnicities (multiracial to Asian), and medical departments (intensive care unit to general ward).

## Methods

### Overview

**Figure 1:**
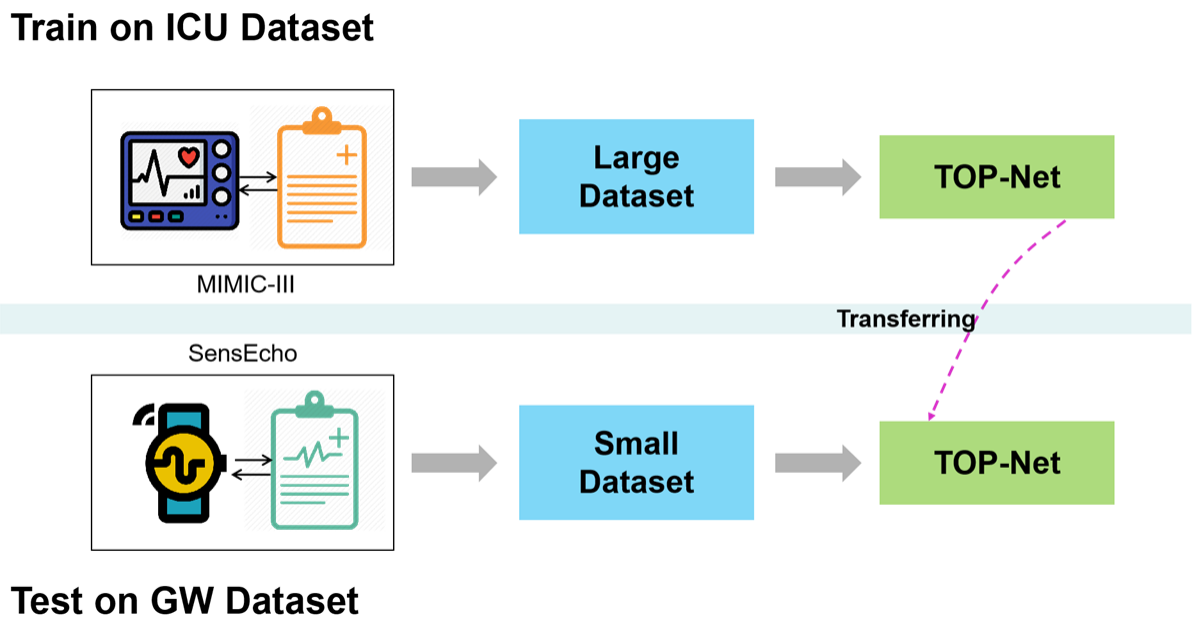
The process of developing and transferring the early tachycardia onset model, TOP-Net. GW: general ward; ICU: intensive care unit.

We leveraged a large data set from the Medical Information Mart for Intensive Care III (MIMIC-III) [24] and its matched physiological waveform database (recorded with monitors) [36] to develop the TOP-Net model (codes available [37]). The pretrained model was transferred to a relatively small data set, from patients who were continuously monitored with a medical-grade wearable embedded system (SensEcho, Beijing SensEcho Science & Technology Co Ltd) in a real clinical environment [38]. The process is presented in Figure 1.

### Methodology

We combined 2 types of data to develop TOP-Net: (1) information from biological sensors (wearable), including heart rate, respiratory rate and SpO_2_; (2) patients’ personal information from electronic health records, which represents their individual health status when admitted to the hospital, including age, gender, admission type, first care unit, and history of cardiovascular disease.

### TOP-Net Tachycardia Onset Early Prediction Using BiLSTM Model

#### Model Overview

**Figure 2:**
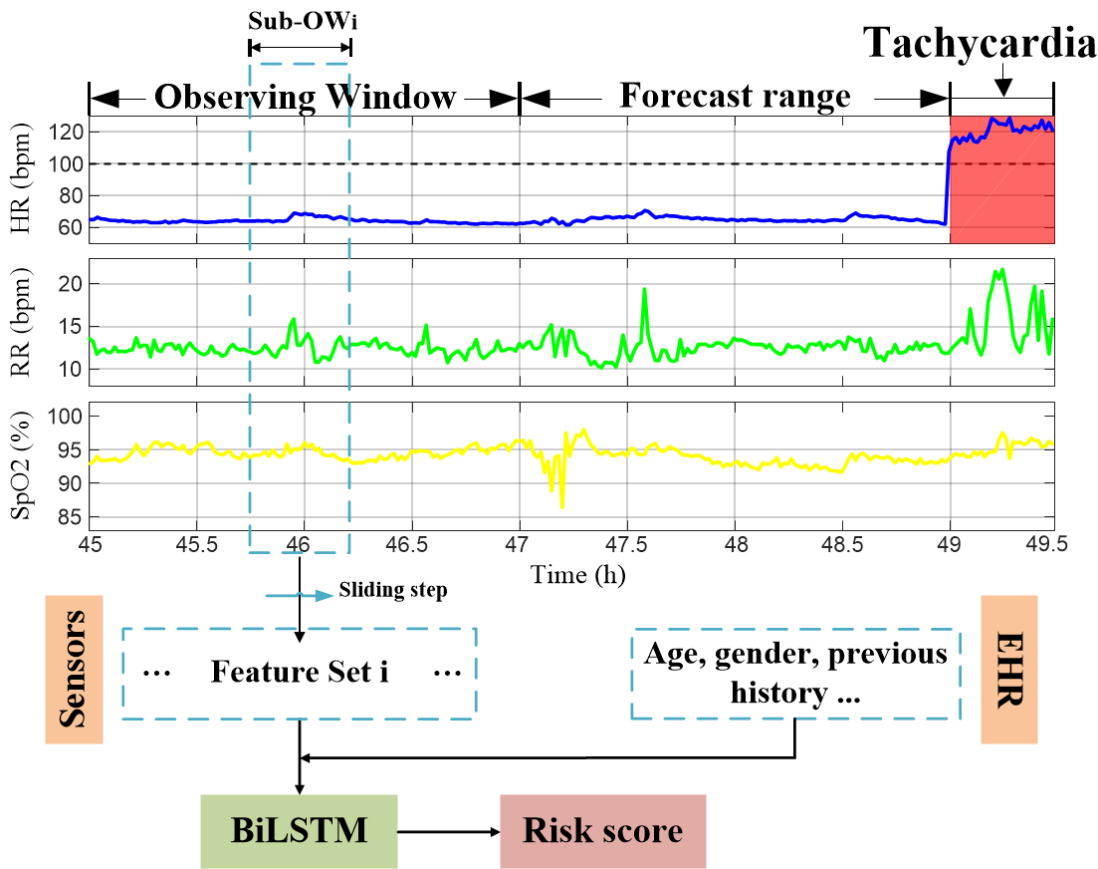
An overview of TOP-Net using the cohort admission and personal measurement data in hospital. BiLSTM: bidirectional long short-term memory; EHR: electronic health record; HR: heart rate; RR: respiratory rate; SpO_2_: blood oxygen saturation.

BiLSTM [39], a sequential model, can capture the complex and multivariate dynamics in longitudinal electronic health record data and continuously collected physiological signals that is typically used in acute condition prediction, classification, and subphenotype identification [40]. We developed the model (Figure 2) using BiLSTM to take advantage of potential long-term and short-term changes and associated characteristics of physiological state.

#### Step 1: Calculate Statistical Features

We used a BiLSTM algorithm to represent the relationship between the multiple timeseries collected by biological sensors. Data from an observing window before tachycardia onset were used to train the model. Inspired by convolutional-LSTM model [41], we designed the model to use the statistical features of the raw timeseries signals as inputs within a sliding sub–observing window. The results for all sub–observing windows were concatenated along the time and fed into the model.

We explored 8 types of statistical features—mean, standard deviance, slope, quantiles, sum, absolute energy (ƒ_1_), aggregation function of autocorrelation (ƒ_2_), and measurement of discrimination power (ƒ_3_)—that are commonly used to describe the timeseries characteristics. Herein, we focus on explaining the calculation process of ƒ_1_, ƒ_2_, and ƒ_3_.

The absolute energy of the timeseries is calculated as


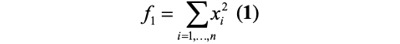


The correlation of a timeseries and its time lag is described by ƒ_2_,


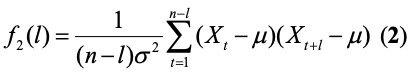


which is a similarity measurement index where *X*_i_ is a timeseries value at one time point, *n* is the length of *X*, σ^2^ and μ are estimations of the timeseries variance and mean, respectively, and *l* is the time lag [42].

The nonlinearity of a timeseries is quantized using


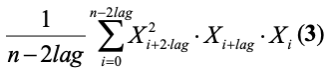


where *lag* is a time delay operator (equal to *l*) [43].

#### Step 2: Fuse Patient Characteristics

We extracted the previously mentioned static patient information which was merged with the statistical features. The concatenated vectors were normalized and input to the BiLSTM model.

#### Step 3: Obtain Tachycardia Onset Risk Score

In this step, TOP-Net determines a real-time risk score that evaluates an individual risk probability of tachycardia onset. When the risk score continuously exceeds the threshold set by the doctor for a period of time, the caregiver is alerted.

### Medical Information Mart for Intensive Care (MIMIC)

**Figure 3:**
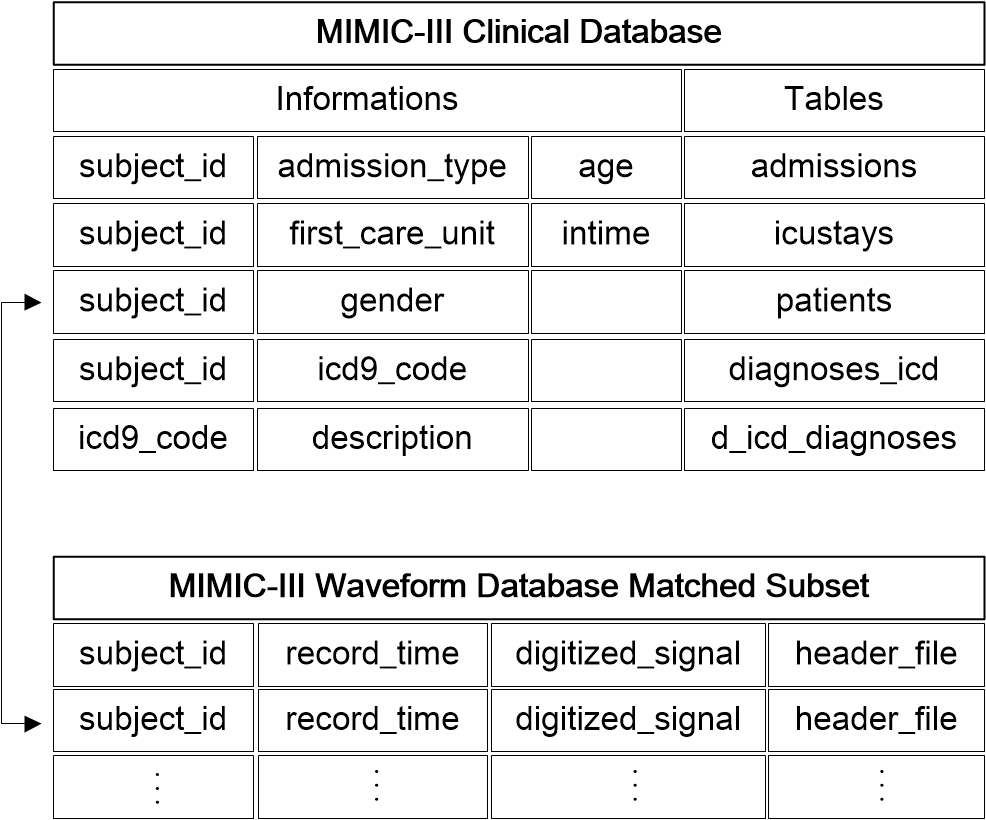
The connection between clinical and waveform information in the MIMIC-III database.

MIMIC III is a large, publicly available critical care database (version 1.4 [24]), with 38,557 adult patients’ (52,955 ICU admissions) detailed hospital information such as demographic information, laboratory test results, and diagnosis codes. Patients’ multiple physiological signals (waveforms) and corresponding numeric format of vital signs are stored in the MIMIC III Waveform Database, which contains 10,282 patients’ time alignment information and 22,247 numeric records that can be matched to the clinical database [36]. The basic information is stored in the tables of *admissions*, patients’ hospital admission information; *icustays*, ICU transfer (in and out) information; *patients*, individual birth and death dates; and *diagnoses_icd*, diagnosis codes during hospitalization. All of the tables can be associated with *subject_id*, a unique identity of patients. The waveform database includes the header files (name, unit, and recording frequency) and segments of recordings (numeric signals). Figure 3 presents the method used to link tables of information with the temporal waveforms.

### Continuous Monitoring Database for the General Ward

**Figure 4:**
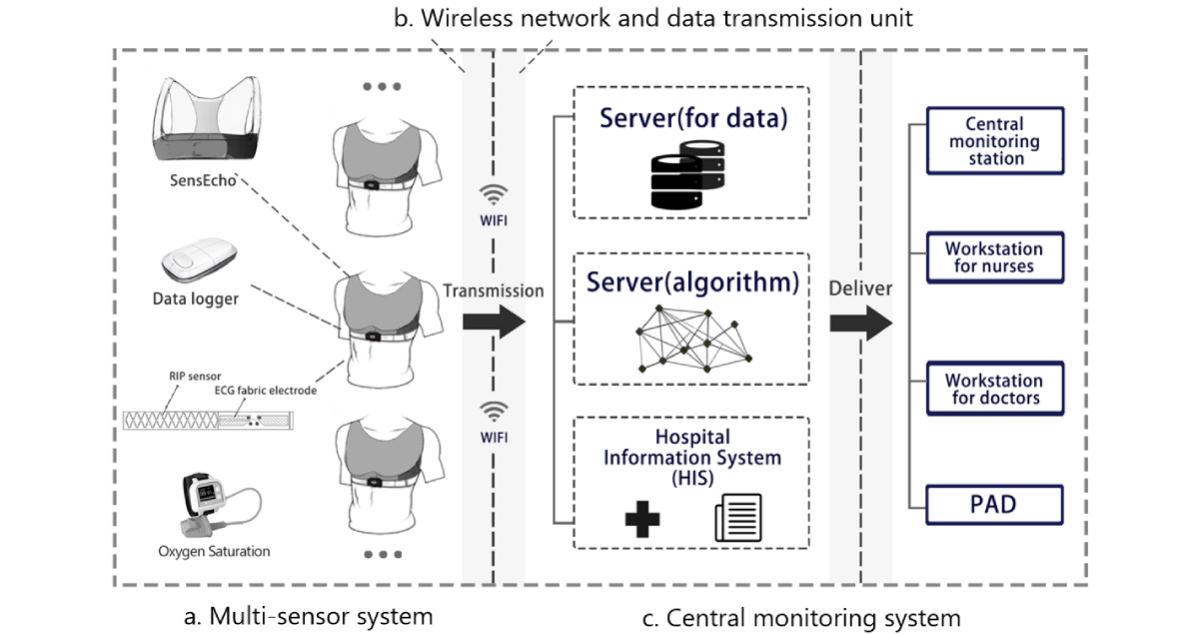
Overview of the SensEcho system.

The use of general ward data was approved by the ethics committee of the General Hospital of PLA (S2018-095-01). In the general ward, we utilized a SensEcho medical-grade monitoring system, which can monitor patients anytime and anywhere. SensEcho contains 3 parts (Figure 4): a wearable multisensor system unit, a wireless network and data transmission unit, and a central monitoring system [35,38]. The multisensors include a single-lead ECG sensor (200 Hz), a sensor for respiratory inductive plethysmography (25 Hz), a noninvasive photoplethysmogram sensor for SpO_2_ monitoring (1 Hz) based on near-infrared spectroscopy, and a posture recognition sensor using a 3-axis accelerometer. These signals are collected and stored in a data logger. The logger has an ultra–low power Wi-Fi module and supports long-term data transmission by relying upon hospital networks. The central monitoring system receives information, processes data, and delivers and displays information. The algorithms deployed on the system included signal quality evaluation, signal processing, real-time abnormal event monitoring and early prediction, and patients’ health assessment, which were packaged as a toolkit (Midas). The accuracy, stability, and effectiveness of our system have been validated in previous studies [44-46].

**Figure 5:**
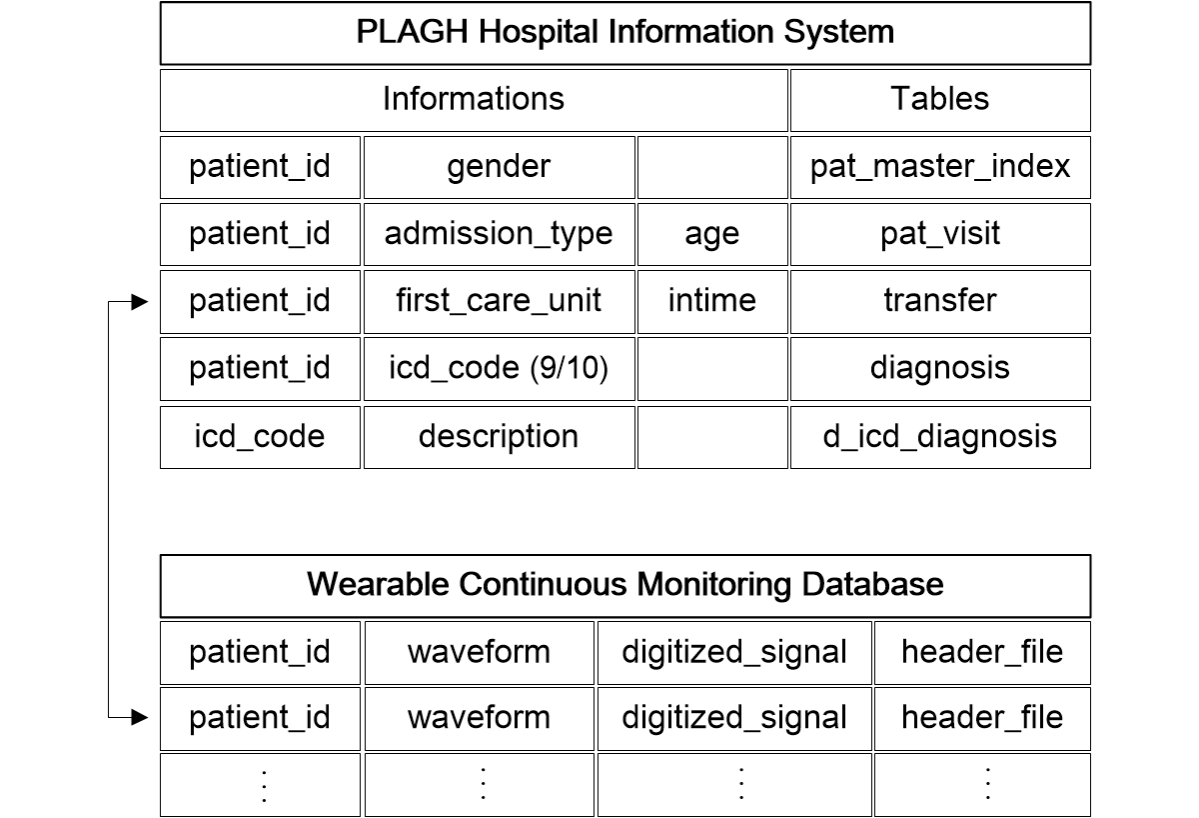
The connection between clinical and waveform information monitored by SensEcho.

Patients admitted to the hospital were assessed by a doctor using the system. Continuous monitoring physiological signals were transmitted to the hospital server and the data in numeric format were acquired based on the waveform processing function in Midas. The clinical information was stored separately in the hospital information system. Data from the different sources were linked (Figure 5) using *patient_id*, a unique identification of patients similar to *subject_id* in MIMIC III.

### Tachycardia Onset Diagnostic Criteria

Diagnostic tachycardia onset criteria were determined by 3 clinical experts from the Emergency Department, the general ward, and surgical ICU. A tachycardia event was defined as any of the following: (1) heart rate above 100 bpm sustained over 30 minutes; (2) heart rate above 130 bpm sustained over 20 minutes; (3) heart rate above 150 bpm sustained over 5 minutes. The initial timepoint meeting of any of these conditions was recognized as tachycardia onset.

### Experiments

#### Data Set

In the ICU environment, we selected 5699 patients with the following criteria: age over 18 years old, admitted to the hospital and ICU for the first time, monitoring data longer than 14 hours with heart rate, respiratory rate, and SpO_2_ recordings. The size of the observing window was chosen as 2 hours, which was used to extract the statistical features. The negative sample set was built by extracting information in the observing window with a 1-hour sliding step throughout monitoring for patients without tachycardia. The positive sample set was acquired by selecting the same features in the observing window before the occurrence of tachycardia with a forecast range. To balance the ratio of positive and negative samples, we kept extracting positive samples with a 5-minute delay based on the former (for target replication), which is a method used in a previous study [47]. The data were downsampled from per second to per minute by averaging. If more than 30% were null or 0 values of all variables at a certain time, the missing values were filled using the forward interpolation method. We randomly picked the number of negative samples close to the positive samples to further decrease class imbalances. There were 2748 and 2130 negative and positive samples, respectively.

**Figure 6:**
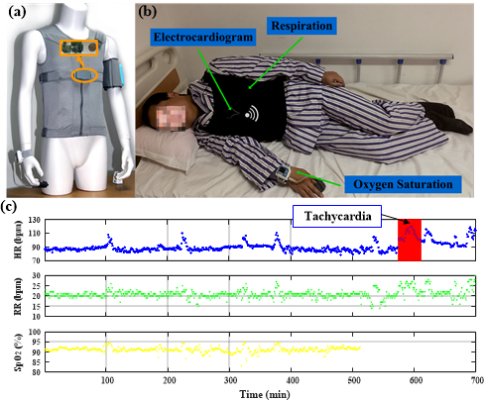
Continuous monitoring using (a) SensEcho system with (b) example of a patient with sensors attached, and (c) sample data. HR: heart rate; RR: respiratory rate; SpO_2_: blood oxygen saturation.

In the general ward, we deployed the wearable grade monitoring system (Figure 6a) in a cardiovascular disease department in January 2018. We collected data from 367 patients for research. The inclusion criteria for monitoring duration was reduced to from 14 hours to 4 hours to take into account patient length of stay. A total of 259 patients were included, and 2300 negative samples and 270 positive samples were extracted. Figure 6b shows a patient wearing a multisensor shirt, and Figure 6c shows an example of a patient encountering tachycardia.

#### Developing the Prediction Model

In the early prediction model, developed from the MIMIC-III data set, predictions (forecast ranges) with TOP-Net were explored from 0 hour to 6 hours with a 2-hour interval. A total of 21 statistical features were included (Table 1). The size of sub–observing window and sliding step were individually set to 20 minutes and 5 minutes, respectively. We calculated all statistical values in sub–observing windows, sequentially amalgamated, and fed them into the model. The data set was randomly split to 80% of the training set and 20% of the testing set according to the patient’s hospitalization number. The 5-fold cross-validation together with random search was used to tune the hyperparameters based on the training set considering the sample size [48]. The hidden size was set to 32. We tested learning rates ranging from 1^-4^ to 1^-2^ with an interval of 1^-4^ and training epochs from 5 to 100 with an interval of 10. The best hyperparameters were determined by minimizing validation loss. We retrained the model using the optimal hyperparameters on the training set, and the performance of the model was assessed on the test set.

**Table 1:** Statistical features constructed in this study.

Feature type and name		Feature description
**Heart rate (n=10)**		
	hr_mean	Mean heart rate
	hr_std	Heart rate SD
	hr_sum	Sum of heart rate
	hr_slope	Slope of heart rate
	hr_abs_energy	ƒ_1_ of heart rate
	hr_c2	ƒ_3_ of heart rate with *lag*=2
	hr_c3	ƒ_3_ of heart rate with *lag*=3
	hr_quantiles_01	10% quantile of heart rate
	hr_quantiles_03	30% quantile of heart rate
	hr_quantiles_07	70% quantile of heart rate
**Respiratory rate (n=5)**		
	resp_mean	Mean respiration rate
	resp_std	Respiration rate SD
	resp_slope	Slope of respiration rate
	resp_abs_energy	ƒ_1_ of respiration rate
	resp_c3	ƒ_3_ of respiration rate with *lag*=3
**SpO_2_^a^ (n=5)**		
	spo2_mean	Mean SpO_2_
	spo2_std	SD of SpO_2_
	spo2_slope	Slope of SpO_2_
	spo2_c3	ƒ_3_ of SpO_2_ with *lag*=3
	spo2_abs_energy	ƒ_1_ of SpO_2_
**Together (heart rate, respiratory rate, SpO_2_) (n=1)**		
	all_autocorrelation	Mean value of ƒ_2_ using all vital signs with the default *l*=40

^a^SpO_2:_ blood oxygen saturation.

### Comparison With Baseline Models

To further investigate the performance of TOP-Net, we designed subexperiments 1, 2, and 3 to obtain a comprehensive assessment. In subexperiment 1, the model was acquired without considering personal information and bidirection memory functions. That is, LSTM and convolutional neural network models were obtained in a total cohort without considering the personal information of patients. The structure of the LSTM was consistent with that of a BiLSTM, and the convolutional neural network model had 2 convolutional layers. In subexperiment 2, conventional machine learning methods, including extreme gradient boosting [49], multilayer perceptron, and random forest, were compared with TOP-Net with default model parameters. In subexperiment 3, different feature combinations were examined: (1) all vital signs, (2) heart rate, (3) heart rate and respiratory rate, and (4) heart rate and SpO_2_.

### Performance Evaluation Metrics

Prediction performance was measured with 6 metrics: sensitivity, specificity, accuracy, F1 score, precision, and area under the receiver operating characteristic curve (AUROC).

### Model Validation and Transfer to the General Ward

The performance of TOP-Net was validated using the data collected in the general ward (small data set obtained within 1 year) by the SensEcho system. A transferrable model suitable for non-ICU patients was acquired by finetuning the ICU scenario model. The model performance was also assessed with the 6 metrics using 5-fold cross-validation due to the small sample size.

### Experimental Platform

We utilized PostgreSQL (version 9.6; PostgreSQL Global Development Group) to extract the clinical data. All data processing and analyses, model development, and result visualization was performed with Python (version 3.7.1) and CUDA (version 10.0).

## Results

### Data Sets

Table 2 shows admission information summary statistics for the study cohorts. The patients’ ages were slightly higher in the ICU cohort and most of them were admitted to the hospital for emergencies. A large proportion of patients were admitted for elective reasons in the cardiovascular disease department of our hospital. Furthermore, a higher proportion of patients had a history of cardiovascular diseases in the general ward.

**Table 2:** Study cohorts.

		ICU^a^ cohort (n=5699)	General ward cohort (n=259)
Age (years), median (IQR)		66.15 (53.97, 77.78)	61.00 (53.00, 67.50)
**Gender, n (%)**			
	Female	3262 (57.2)	105 (40.5)
	Male	2437 (42.8)	154 (59.5)
**Admission type, n (%)**			
	Elective	979 (17.2)	227 (87.6)
	Emergency	4550 (79.8)	32 (12.4)
	Urgent	170 (3.0)	—^b^
**First care unit, n (%)**			
	Coronary care	1190 (20.9)	—
	Cardiac surgery recovery	1118 (19.6)	—
	Medical ICU	1501 (26.3)	—
	Surgical ICU	1320 (23.2)	—
	Trauma/surgical ICU	570 (10.0)	—
Cardiovascular diseases, n (%)		4933 (86.6)	234 (90.3)

^a^ICU: intensive care unit.

^b^No data.

### Model Performance

#### Evaluation Based on the ICU Cohort

**Figure 7:**
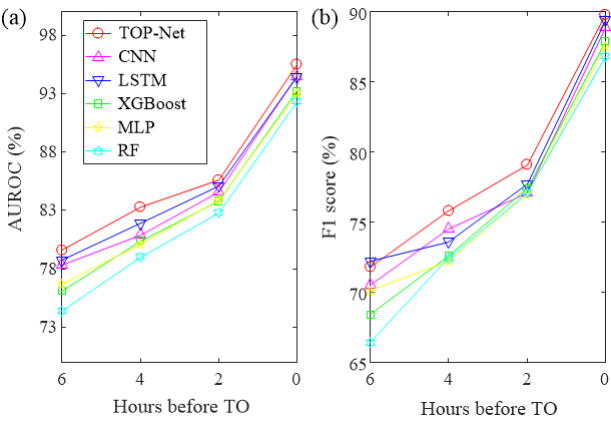
TOP-Net performance: (a) AUROC and (b) F1 score. AUROC: area under the receiver operating characteristic curve; CNN: convolutional neural network; LSTM: long short-term memory; XGBoost: extreme gradient boosting; MLP: multilayer perceptron; RF: random forest; TO: tachycardia onset.

We leveraged 5-fold cross-validation to select optimal hyperparameters with the training set and assessed the performance of the model on the test set. The hyperparameter values that we selected were learning rate =0.0002, epoch=20, and batch size=64. Figure 7 and Table 3 summarize the results from subexperiment 1 and subexperiment 2. The AUROC and F1 score for TOP-Net were consistently better than those of other models, with the exception of F1 score (TOP-Net’s was slightly lower than that of the LSTM model for 6 hours prediction, though TOP-Net’s sensitivity was slightly higher than of the LSTM at this time).

Although the 95% CI in subexperiment 1 overlaps, TOP-Net has better performance than LSTM and convolutional neural network in each prediction range above 0.5%-1%. Therefore, fusing patient personal information and bidirection memory makes the prediction model more accurate and robust. In subexperiment 2, TOP-Net was consistently superior to the other machine learning models, especially 6 hours before tachycardia onset; TOP-Net performs well (AUROC 0.796, 95% CI 0.768-0.824; sensitivity 0.753, 95% CI 0.663-0.793; specificity 0.720, 95% CI 0.645-0.758; and F1 score 0.718).

**Figure 8:**
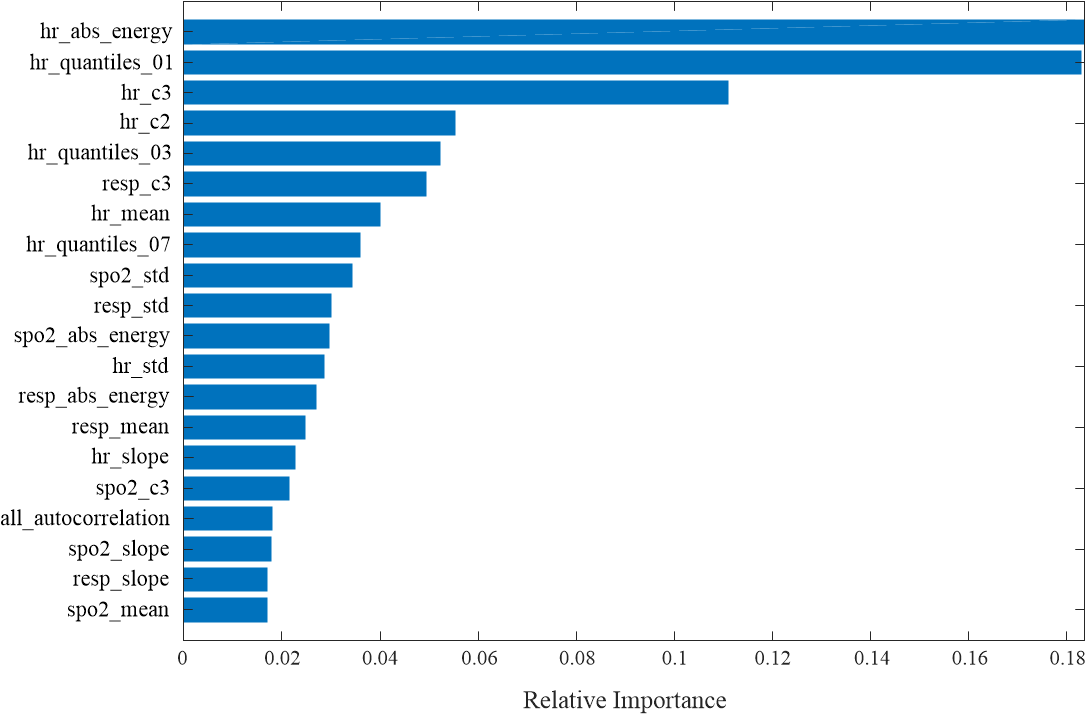
Statistical feature rankings.

In Table 4, the results for models using heart rate (n=10), heart rate and respiratory rate (n=15), heart rate and SpO_2_ (n=15), and statistical features of all vital signs (n=21) are shown. For 2- to 6-hour forecast ranges the model with all of the features input has the best performance with highest AUROC values. The performance is slightly reduced when inputting heart rate and respiratory rate, or heart rate and SpO_2_. The performance was the worst when including only heart rate statistical features. The statistical characteristics of heart rate play a dominant role in real-time diagnosis. Furthermore, we employed the extreme gradient boosting algorithm to rank the importance of 21 designed features for a forecast range of 6 hours. The top 8 features (Figure 8) were *hr_abs_energy*, *hr_quantiles_01*, *hr_c3*, *hr_c2*, *hr_quantiles_03*, *resp_c3*, *hr_mean*, and *hr_quantiles_*07. The nonlinearity features—*hr_c3* and *hr_c2* (ƒ_3_ with *lag*=3 and *lag*=2)—were ranked third and fourth, respectively. The respiratory feature *resp_c3* was ranked sixth.

**Table 3:** The detailed information of performance comparison (TOP-Net vs other models).

Forecast range and model			AUROC^a^ (%) (95% CI)		Accuracy (%)		Sensitivity (%) (95% CI)		Specificity (%) (95% CI)		F1 score (%)		Precision (%)
**0 hours**													
	TOP-Net	95.5 (94.2-96.8)		90.1		89.1 (81.9-91.8)		92.1 (85.9-94.3)		89.8		90.5	
	CNN^b^	94.5 (93.0-96.0)		89.3		85.9 (80.7-89.4)		92.3 (87.0-95.1)		88.9		92.1	
	LSTM^c^	94.4 (92.9-96.0)		89.8		88.9 (83.9-91.8)		90.8 (81.1-93.8)		89.4		89.9	
	XGBoost^d^	93.2 (91.5-94.9)		88.3		81.9 (75.7-85.6)		93.0 (87.4-96.0)		87.9		94.9	
	MLP^e^	93.0 (91.3-94.8)		87.9		85.6 (80.2-88.9)		89.9 (84.6-93.2)		87.5		89.5	
	Random forest	92.3 (90.5-94.2)		87.3		85.1 (80.2-88.8)		89.0 (82.1-92.8)		86.8		88.6	
**2 hours**													
	TOP-Net	85.6 (83.2-88.0)		79.6		77.6 (70.8-81.3)		81.6 (74.2-85.1)		79.1		80.6	
	CNN	84.6 (82.1-87.1)		77.6		78.6 (71.3-83.2)		77.8 (71.2-81.4)		77.1		75.6	
	LSTM	85.1 (82.7-87.5)		78.2		88.6 (81.0-92.0)		67.4 (56.8-71.5)		77.7		76.8	
	XGBoost	83.8 (81.2-86.3)		78.0		74.5 (66.7-79.1)		80.9 (73.9-84.5)		77.4		80.5	
	MLP	83.9 (81.4-86.4)		77.5		78.3 (71.3-82.2)		77.7 (69.9-82.0)		77.0		75.8	
	Random forest	82.8 (80.2-85.4)		77.7		71.5 (63.6-76.6)		82.3 (76.4-86.0)		77.1		83.7	
**4 hours**													
	TOP-Net	83.3 (80.7-85.8)		76.3		83.5 (75.5-85.9)		72.2 (63.8-74.7)		75.8		69.4	
	CNN	80.9 (78.2-83.7)		75.2		71.5 (63.6-76.3)		78.8 (70.0-82.5)		74.5		77.8	
	LSTM	81.9 (79.2-84.5)		74.2		73.1 (65.5-77.9)		76.3 (69.6-80.1)		73.6		74.1	
	XGBoost	80.4 (77.7-83.2)		73.4		68.1 (60.2-72.7)		78.5 (72.0-82.8)		72.6		77.8	
	MLP	80.1 (77.3-82.8)		72.9		73.9 (66.9-78.7)		72.0 (65.1-76.3)		72.2		70.6	
	Random forest	79.0 (76.1-81.9)		73.3		64.5 (60.6-71.4)		79.9 (73.4-84.8)		72.4		82.5	
**6 hours**													
	TOP-Net	79.6 (76.8-82.4)		72.1		75.3 (66.3-79.3)		72.0 (64.5-75.8)		71.8		68.6	
	CNN	78.3 (75.4-81.1)		70.9		79.3 (72.8-83.7)		64.1 (57.1-69.1)		70.5		63.5	
	LSTM	78.7 (75.9-81.5)		72.5		74.0 (67.0-78.4)		71.8 (64.1-76.0)		72.2		70.5	
	XGBoost	76.1 (73.1-79.0)		69.1		76.3 (69.5-75.4)		64.1 (55.1-68.9)		68.4		62.0	
	MLP	76.7 (73.8-79.6)		70.6		71.9 (65.1-76.7)		69.4 (61.7-74.5)		70.1		68.4	
	Random forest	74.4 (71.4-77.5)		67.2		69.1 (59.0-74.3)		66.7 (59.3-70.6)		66.4		63.9	

^a^AUROC: area under the receiver operating characteristic curve.

^b^CNN: convolutional neural network.

^c^LSTM: long short-term memory.

^d^XGBoost: extreme gradient boosting.

^e^MLP: multilayer perceptron.

**Table 4:** Performance of TOP-Net with the different types of features.

Forecast range and feature type			AUROC^a^ (%) (95% CI)		Accuracy (%)		Sensitivity (%) (95% CI)		Specificity (%) (95% CI)		F1 score (%)		Precision (%)
**0 hours**													
	All	95.5 (94.2-96.8)		90.1		89.1 (81.9-91.8)		92.1 (85.9-94.3)		89.8		90.5	
	HR^b^+SpO_2_^c^	95.2 (93.8-96.6)		90.4		89.4 (84.7-93.1)		91.9 (85.9-94.1)		90.1		90.8	
	HR+RR^d^	95.3 (93.9-96.7)		90.0		89.6 (84.7-92.3)		91.0 (84.8-94.3)		89.6		89.6	
	HR	95.5 (94.2-96.9)		90.1		89.1 (83.9-92.3)		92.1 (86.5-94.9)		89.8		80.6	
**2 hours**													
	All	85.6 (83.2-88.0)		79.6		77.6 (70.8-81.3)		81.6 (74.2-85.1)		79.1		80.6	
	HR+SpO_2_	83.3 (80.8-85.9)		76.9		77.1 (70.6-81.0)		76.4 (69.4-80.4)		76.1		75.1	
	HR+RR	84.4 (81.9-86.9)		79.1		75.4 (69.1-79.3)		82.0 (75.3-86.5)		78.6		82.1	
	HR	82.9 (80.3-85.5)		76.9		78.6 (71.8-82.7)		73.9 (66.7-78.0)		76.3		74.1	
**4 hours**													
	All	83.3 (80.7-85.8)		76.3		83.5 (75.5-85.9)		72.2 (63.8-74.7)		75.8		69.4	
	HR+SpO_2_	82.3 (79.6-84.9)		75.8		76.5 (70.0-81.3)		74.9 (67.6-79.2)		75.0		73.6	
	HR+RR	82.1 (79.5-84.8)		75.6		72.7 (66.4-77.7)		77.9 (70.0-82.3)		75.0		77.5	
	HR	80.4 (77.6-83.2)		73.6		75.5 (67.2-79.9)		72.7 (66.0-77.0)		72.9		70.5	
**6 hours**													
	All	79.6 (76.8-82.4)		72.1		75.3 (66.3-79.3)		72.0 (64.5-75.8)		71.8		68.6	
	HR+SpO_2_	77.6 (74.7-80.5)		71.9		70.0 (62.6-74.4)		74.5 (66.5-78.6)		71.5		73.0	
	HR+RR	78.7 (75.8-81.5)		72.0		78.8 (71.6-83.3)		67.6 (61.2-72.0)		71.6		65.6	
	HR	75.5 (72.5-78.6)		70.0		67.2 (59.5-72.1)		73.3 (66.9-77.7)		69.4		71.7	

^a^AUROC: area under the receiver operating characteristic curve.

^b^HR: heart rate.

^c^SpO_2_: blood oxygen saturation.

^d^RR: respiration rate.

#### Model Validation in the General Ward

We assessed the performance of the model 2 hours before tachycardia onset because the interval between the tachycardia onset and the admission time to the department was short in our scenario of the general ward. Given the limited training data, we used the transfer learning method to finetune the model. The parameters were learning rate=0.0002, epoch=18, and batch size=32. The 5-fold cross-validation was also used to assess the performance and prevent possible overfitting. The retraining results can be seen in Table 5. TOP-Net had a stable outcome and outperformed the other 5 models (AUROC 0.965, accuracy 0.937, sensitivity 0.955, specificity 0.881, F1 score 0.793, and precision 0.680. Compared with the model in ICU, the difference in prediction performance might be caused by the difference in the severity of the patient’s disease. Although convolutional neural network’s F1 score was much higher, its sensitivity, to which clinicians pay more attention, was lower than that of TOP-Net.

**Figure 9:**
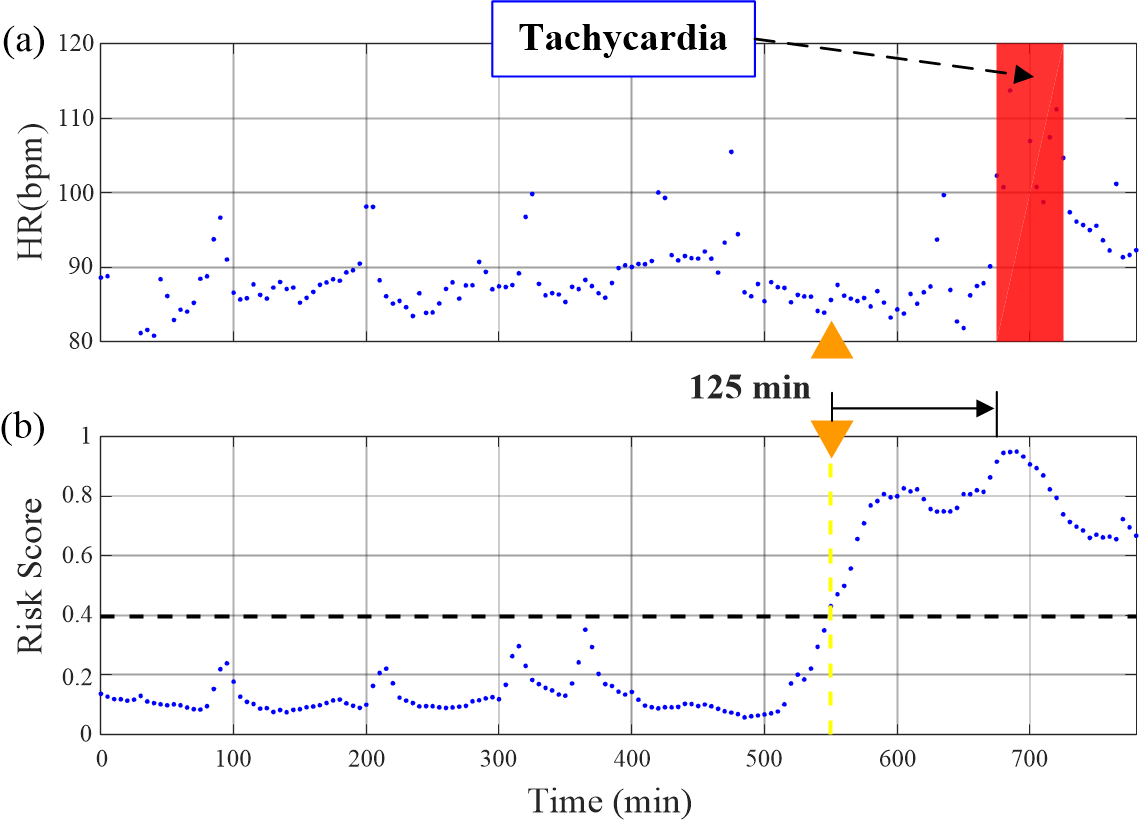
Example of a tachycardia event and our risk score of predicting tachycardia onset. HR: heart rate.

Figure 9 shows real-time risk scores of tachycardia onset and an example of early tachycardia onset prediction with TOP-Net. In Figure 9a, the patient encountered a tachycardia event after admission from 675 to 725 minutes. The risk probability was assessed every 5 minutes; Figure 9b presents real-time risk. We set the alarm threshold to 0.40 with a trade-off predictive effect of sensitivity and specificity. The risk score begins to rise after the 555th minute, showing that our model can predict the tachycardia event 125 minutes beforehand.

**Table 5:** TOP-Net performance based on transfer learning in the general ward (2-hour forecast range).

Model	AUROC^a^, mean (SD)	Accuracy (%), mean (SD)	Sensitivity (%), mean (SD)	Specificity (%), mean (SD)	F1 score (%), mean (SD)	Precision (%), mean (SD)
TOP-Net	96.5 (1.92)	93.7 (1.02)	95.5 (4.85)	88.1 (4.28)	79.3 (4.33)	68.0 (5.99)
CNN^b^	93.8 (2.02)	95.3 (1.43)	90.1 (2.88)	88.1 (8.4)	83.8 (5.38)	78.8 (9.85)
LSTM^c^	93.2 (1.89)	92.6 (0.61)	93.6 (2.76)	81.5 (5.6)	73.0 (3.4)	60.0 (4.89)
XGBoost^d^	89.9 (2.1)	92.9 (1.1)	83.4 (5.2)	82.6 (7.9)	73.7 (3.7)	66.6 (6.8)
MLP^e^	84.2 (4.1)	91.0 (0.7)	75.9 (9.6)	78.9 (9.1)	62.6 (2.0)	54.0 (2.9)
Random forest	87.3 (3.0)	92.5 (1.0)	76.6 (5.2)	86.8 (4.7)	75.0 (3.7)	73.8 (4.9)

^a^AUROC: area under the receiver operating characteristic curve.

^b^CNN: convolutional neural network.

^c^LSTM: long short-term memory.

^d^XGBoost: extreme gradient boosting.

^e^MLP: multilayer perceptron.

## Discussion

### General

In this study, we developed a model using a publicly accessible data set and transferred it to a real clinical scenario. The performance of TOP-Net for predicting tachycardia onset 0 to 6 hours in advance was better than that of the baseline models (timeseries prognosis methods and conventional machine learning methods without timing characteristics); TOP-Net outperformed benchmarks of 2 deep learning models, 2 ensemble, and 1 neural network models for predictions 6 hour in advance.

Many continuous monitoring physiological status studies have indicated the deterioration of vital signs occurred more than 6 to 12 hours before serious adverse events [50]. Continuous monitoring, early prediction, and intervention tachycardia can reduce the occurrence of heart failure, cardiac arrest, and death. This paper proposed TOP-Net, a tachycardia onset early prediction model leveraging the BiLSTM algorithm with 8 easily accessible vital signs and personal information. TOP-Net was trained using a large ICU data set and transferred to the general ward scenario with patients monitored by wearable sensors. TOP-Net has been validated to be consistently superior to the baseline models when predicting tachycardia onset from 0 to 6 hours in advance. Including patient characteristics allowed more accurate tachycardia onset prediction than those by other models without this information. Moreover, TOP-Net achieved forecasting tachycardia onset 6 hours beforehand, and the transferred model also performed well in our clinical scenario.

In recent years, some novel models for early risk prediction of adverse events have been developed based on electronic health records or physiological signals. Pan et al [51] utilized a self-correcting deep learning approach to predict whether acute kidney injury would occur in a subsequent 6 hours. Futoma et al [52] developed a multitask Gaussian process recurrent neural network classifier to early detect sepsis achieving 4 hours in advance. Tonekaboni et al [53] trained a convolutional neural network and LSTM fusion model to predict cardiac arrest from physiological signals 24 hours in advance. For tachycardia onset prediction, Lee et al [17] used an artificial neural network–based model and 104 samples to predict ventricular tachycardia 1-hour before occurrence. Yoon et al [54] adopted a random forest–based model and 1494 samples achieving detection 75 minutes in advance. Our real-time prediction model, using the deep neural architecture on 4878 sample sets, demonstrated better and more robust performance than those of multiple baseline models, which included artificial neural network and random forest models, when predicting tachycardia onset 0 to more than 6 hours beforehand.

It is necessary for clinicians to combine a patient’s current symptoms, basic information, and past medical history to diagnose disease severity [55]. For example, the proportion who might have cardiovascular disease and the risk of sustained high heart rate is not the same for patients of different ages with different histories of disease. This useful information is usually recorded in electronic health records. Recently, several researchers have tried to combine the analysis of 2 kinds of materials to represent comprehensive information and improve the performance of the models: Xu et al [56] proposed a model to predict physiological decompensation and length of ICU stay by analyzing ECG and medical records data, and Nemati et al [57] employed high-resolution vital signs and electronic health records to achieve early sepsis prediction. However, little attention has been paid to tachycardia prognosis. In this paper, we integrated electronic health record and biosensor data to accomplish early prediction. The results of subexperiment 1 show that fusing electronic health record information can improve the accuracy of early prediction compared with the LSTM and convolutional neural network models.

Risk prediction is a core task in the artificial intelligence–assisted medical domain. Cardiovascular disease prediction models based on electronic health record analysis have been studied [58-60]. Doctor AI [58] requires diagnosis codes, medication codes, or procedure codes to achieve multilabel predictions including heart failure. Jin et al [60] utilized 1864 diagnostic events to train a sequential model to predict the risk of heart failure but because they were limited by the need to obtain more information, the model cannot be used in hospitals with low information integration or in homes. Deep learning models using ECG signals have also been used for predictive health care tasks [61]. While ECG signals are susceptible to interference from physical artifacts, sensors can obtain heart rate using photoplethysmography instead of ECG signals. Therefore, models based on core vital signs can easily be used and to improve prediction performance. We selected 3 vital signs and 5 types of personal information that can easily be acquired from wearable sensors and hospital information systems, respectively. TOP-Net was developed using a large data set and transferred to our actual demand scenario. The results show that it has the potential to be used in ICU and the general ward, which also can be extended to home use. Table 6 presents a comparison between TOP-Net and other state-of-the-art approaches based on input information, model types, scenario for evaluating the model, sample sizes, and performance.

**Table 6:** Review of the performance of related algorithms.

Reference	Information	Model types	Scenario	Sample sizes	Performance
Lee et al 2016 [17]	High-frequency vital signs (1)	Nontemporal, classic machine learning	ICU^a^	52 (positive records); 52 (negative records)	1 hour before ventricular tachycardia: sensitivity 88%; specificity 82%; AUROC^b^ 93%
Forkan et al 2017 [16]	High-frequency vital signs (6)	Nontemporal, classic machine learning	ICU	4893 (positive and negative records)	1-2 hours before tachycardia onset: accuracy 95.85%
Yoon et al 2019 [54]	High-frequency vital signs (3)	Nontemporal, classic machine learning	ICU	787 (positive records); 707 (negative records)	75 minutes before tachycardia onset: accuracy 84.7%-78.2%; AUROC 92.1%-84.2 %
TOP-Net	High-frequency vital signs (3) and electronic health record data (5)	Temporal, deep learning	ICU and the general ward	2130+270 (positive records); 2748+2300 (negative records)	6 hours before tachycardia onset: accuracy 72.1%; AUROC 79.6%

^a^ICU: intensive care unit.

^b^AUROC: area under the receiver operating characteristic curve.

### Limitations

This study had some limitations. Because SensEcho was deployed in the clinic for only 1 year after our research project began, the limited data collected prevented us from directly developing a general ward model. Moreover, interventions such as beta-blocker medication may affect the occurrence of tachycardia onset and cause it to not be captured by the input features. Electronic health records contain rich information such as laboratory tests, clinical orders, and nursing notes that can characterize a patient’s health status and depict the trajectory of diseases. Further studies involving the integration of multivariate timeseries from electronic health records are expected to improve the prediction performance of tachycardia onset, and more data from the general ward for TOP-Net performance evaluation are required.

### Conclusions

TOP-Net for real-time evaluation and early prediction of the risk of tachycardia onset, which made it possible to achieve an early forecast of tachycardia onset 6 hours in advance with clinically acceptable performance. TOP-Net was assessed using 6 metrics, 3 subexperiments, different prediction times from 0 to 6 hours. The comparison between the TOP-Net and the other 5 approaches (2 deep learning models, 2 ensemble models, and 1 artificial neural network model) showed that TOP-Net was superior to the other models. The model with personal information from electronic health records had better performance than those without. The easily accessible input data of the model (3 vital signs and 5 types of personal information) and the good performance of the transferred model in the general ward indicated the early prediction of tachycardia onset using wearable sensors is possible in hospitals or houses.

## Abbreviations

AUROC: area under the receiver operating characteristic curve
BiLSTM: bidirectional long short-term memory
ECG: electrocardiogram
ICU: intensive care unit
LSTM: long short-term memory
MIMIC-III: Medical Information Mart for Intensive Care III
SpO_2_: blood oxygen saturation
